# Chinese Herbal Medicine for Weight Management: A Systematic Review and Meta-Analyses of Randomised Controlled Trials

**DOI:** 10.1155/2021/3250723

**Published:** 2021-03-09

**Authors:** Ann Rann Wong, Angela Wei Hong Yang, Kangxiao Li, Harsharn Gill, Mingdi Li, George Binh Lenon

**Affiliations:** ^1^School of Health and Biomedical Sciences, RMIT University Bundoora, VIC, Australia; ^2^School of Science, RMIT University, Melbourne VIC, Australia

## Abstract

**Objective:**

This review investigated the effects and safety of Chinese herbal medicine (CHM) formulas on weight management.

**Methods:**

Eighteen databases in English, Chinese, Korean, and Japanese were searched from their inceptions to September 2019. The treatment groups included CHM formulations, and the control included placebo, Western medication (WM), and lifestyle intervention (LI), with or without cointerventions (WM and/or LI). Quality of studies was assessed using Cochrane Collaboration's risk of bias assessment tool. Body weight and body mass index (BMI) were analysed in RevMan v5.4.1 and expressed as mean differences with 95% confidence intervals (CI), while adverse events were expressed as risk ratio with 95% CI.

**Results:**

Thirty-nine RCTs were eligible for qualitative analysis, 34 of which were included in the meta-analyses. The majority of studies had a high or unclear risk of selection, performance, and detection bias. Twenty-five CHM studies involving cointerventions revealed that CHM had significant adjunct effects on body weight and BMI at the end of treatment compared to control. No serious adverse events were reported in the CHM groups.

**Conclusion:**

CHM indicates a promising adjunct to facilitate WM or lifestyle change for weight management. However, methodological barriers such as lack of allocation concealment and double-blinding may have led to challenges in data synthesis. More rigorously designed RCTs involving cointerventions are warranted.

## 1. Introduction

Obesity is defined as excess fat accumulation that may lead to serious health conditions such as type 2 diabetes mellitus, coronary heart disease, ischaemic stroke, and certain types of cancer [1, 2]. It is commonly screened and diagnosed according to the body mass index (BMI), with a World Health Organisation (WHO) cut-off point of 25–29.99 kg/m^2^ for overweight and ≥30 kg/m^2^ for obese [2]. Globally, the prevalence of overweight and obesity almost tripled in the last four decades with nearly 40% of adults currently above their normal healthy weight [3]. Clinically, obesity has been considered as a modern chronic disease, as it is associated with impaired quality of life, instability of mental health, and potentially life-threatening comorbidities [1].

The first-line therapy for weight management recommended by the Australian National Health and Medical Research Council includes caloric restriction and increasing physical activity [4]. These, however, were reported to have low compliance and a stricter regime may be required before significant weight loss can be observed [5]. Current antiobesity medications approved by the US Food and Drug Administration (FDA) for inhibiting fat absorption (e.g., orlistat) were subjected to a high incidence of gastrointestinal adverse events such as nausea, vomiting, abdominal discomfort, diarrhoea, and steatorrhoea. Centrally-acting appetite suppressants including phentermine, lorcaserin, and bupropion may involve cardiovascular risk, vulvulopathy, insomnia, and the development of drug tolerance [6]. Although bariatric surgery for individuals with BMI ≥35 kg/m^2^ has greater weight loss outcomes, its risks of postoperative or late complications cannot be ignored [7]. Consequently, patients seek alternative therapies including Chinese herbal medicine (CHM) for weight management.

Various clinical studies have reported therapeutic effects of several CHM formulations compared to placebo, WM, or LI on weight management [8–10]. However, previous systematic reviews could not draw robust conclusions to confirm the weight loss effects of CHM formulations, as a result of methodological limitations and the inherent heterogeneity in study designs [11, 12]. In 2010, the Consolidated Standards of Reporting Trials (CONSORT) [13] statement has been implemented to address inadequate reporting of randomised controlled trials (RCTs) [13]. Therefore, an update to review articles after CONSORT statement would be crucial, particularly to address methodological concerns from previous systematic reviews and to provide evidence and guidance for future clinical practice in weight management.

## 2. Materials and Methods

This study was guided by the Cochrane Handbook for Systematic Reviews of Interventions v5.1. [14] and reported following the preferred reporting items for systematic reviews and meta-analysis (PRISMA) checklist [15]. The protocol for this systematic review was registered with the International Prospective Register of Systematic Reviews (PROSPERO, CRD42019142276).

### 2.1. Search Strategies

Eighteen databases, including 11 English (AMED, CINAHL, ClinicalTrials.gov, Cochrane Library, EMBASE, Informit, ProQuest, PubMed, SciFinder, SCOPUS, and Web of Science), four Chinese (CNKI, CQVIP, Wanfang data, and SinoMed), two Korean (National Assembly Library and KoreaMed), and one Japanese (Japan Science and Technology Information Aggregator Electronic), were comprehensively sought for relevant articles from their respective inceptions up to 15^th^ April 2019, updated on 30^th^ September 2019. Search terms were overweight, obesity, CHM, RCT, and their synonyms. An example search strategy is provided in Table S1. Alongside to electronic database searches, hand-searching of potential articles was undertaken by referring to the bibliography of review articles retrieved.

### 2.2. Selection Criteria

All human RCTs with or without blinding were considered if they (1) involved adult participants (18+ years) irrespective of gender or ethnic background and were overweight or obese as diagnosed according to the standard cut-off points for body weight, BMI, and/or waist circumference [16]; (2) applied oral administration of CHM formulations consisting two or more herbs in the treatment group; (3) compared CHM treatment with placebo, no treatment, Western medication (WM), or lifestyle intervention (LI, including diet and exercise); or (4) included body weight (kilograms) and/or BMI (kilograms/metre^2^) as outcome measures. Cointervention was allowed as long as the same cointervention was applied in both arms.

Studies were excluded if they (1) were non-RCTs; (2) included nonadult participants; (3) did not specify diagnostic criteria for inclusion of obesity or overweight participants; (4) consisted participants with drug-induced obesity; (5) used a single herb ingredient or extract; (6) performed modifications or included varying doses of CHM in the intervention groups; (7) had inappropriate comparisons; (8) did not specify treatment details including ingredients, dosage, and frequency of CHM or WM administered; or (9) included a majority of herbs not found in the Chinese Pharmacopoeia [17].

### 2.3. Data Extraction

Two reviewers screened the title and abstract of studies based on the eligibility criteria to identify potential RCTs, and full-text was retrieved for further screening. Data from eligible studies were extracted into a spreadsheet to facilitate synthesis. The spreadsheet consisted of the author, year, gender, age, country, sample size, duration, intervention regime, outcome measures, and adverse events. One reviewer performed data entry while another validated the dataset to ensure accurate extraction and appropriate translation. Disagreements were resolved by discussing with a third researcher to achieve consensus.

### 2.4. Risk of Bias Assessment

The risk of bias was appraised by two independent reviewers based on the Cochrane Collaboration's risk of bias assessment tool. Nine domains were evaluated, including random sequence generation (selection bias), allocation concealment (selection bias), blinding of participants and personnel (performance bias), blinding of patient-reported outcomes (detection bias), blinding of outcome-assessor's reported outcomes (detection bias), incomplete outcome data (attrition bias), selective reporting (reporting bias), funding source (other bias), and comparability of baseline data (other bias). Each domain was assigned a “low,” “high,” or “unclear” risk of bias within each included study. Using random sequence generation as an example of selection bias, studies were assessed as “high risk” if randomisation was performed with predictable sequence (e.g., odds or even numbers), “unclear risk” if studies did not report specific randomisation techniques, “low risk” if adequate randomisation of sequence (e.g., computer-generated list) was used. Discrepancies of judgements were resolved by discussing with a third investigator to achieve agreement.

### 2.5. Data Analysis

All continuous data (i.e., body weight and BMI) were presented as mean difference (MD) with 95% confidence interval (CI). The frequency of adverse events was presented as risk ratio (RR) with 95% CI. These parameters were entered into Review Manager (Version 5.4.1, Copenhagen: The Nordic Cochrane Centre, The Cochrane Collaboration, 2012) [18] for data analysis. For studies with low heterogeneity (I^2^ ≤ 50%), fixed effects model was used. For studies with high heterogeneity (I^2^ > 50%), random effects model was adopted. Where possible, subgroup and sensitivity analyses were performed to identify sources of heterogeneity. Publication bias for body weight and BMI outcomes was assessed by the visual inspection funnel plots with pseudo-95% CI limits and quantified by Egger's regression and Begg's correlation tests. Statistical significance was set at a value of *p* < 0.05.

## 3. Results and Discussion

### 3.1. Description of Included Studies

**Figure 1:**
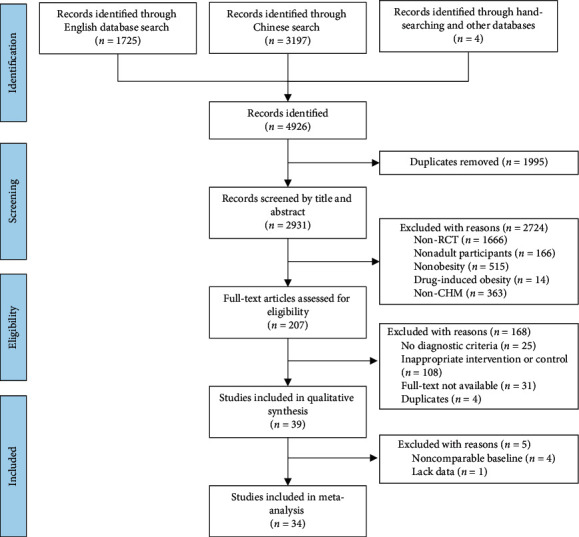
PRISMA flow diagram illustrating selection and exclusion of studies for qualitative review and meta-analysis.

The literature search identified a total of 4926 records and 39 studies were finally included in this review [19–57]. Among them, five studies were excluded from the meta-analyses due to baseline incomparability (*n* = 4) [22, 35, 37, 53] and lack of data (*n* = 1) [19]. The study selection process is illustrated in Figure 1.

All included studies were randomised, parallel-group, controlled trials conducted in China (*n* = 32), Korea (*n* = 4), Australia (*n* = (2), and Japan (*n* = (1) from 2004 to 2019. All studies were two armed except for one study [40] which had three. The treatment duration ranged from one month to six months. A total of 3415 adult participants, aged 18 to 78 years were included in the review. Nineteen studies reported both outcome measures of body weight and BMI, 17 studies reported BMI only, and three studies reported body weight only. The main comparisons identified from the studies were (1) CHM versus placebo (*n* = 6) [22–24, 28, 33, 41], (2) CHM versus WM (*n* = 5) [29, 34, 43, 51, 52], (3) CHM plus LI versus same LI (*n* = 11) [19, 20, 25, 31, 32, 35, 36, 44, 48, 50, 53], (4) CHM plus LI versus placebo plus same LI (*n* = 1) [42], (5) CHM plus LI versus WM plus same LI (*n* = 7) [26, 27, 30, 38, 40, 47, 57], and (6) CHM plus same WM and LI versus same WM and LI (*n* = 9) [21, 37, 39, 45, 46, 49, 54–56]. The characteristics of included studies are detailed in Table 1, arranged according to comparison groups.

- Characteristics: COB, central obesity; HBP, high blood pressure; HLD, hyperlipidaemia; IGT, impaired glucose tolerance; IR, insulin resistance; MET, metabolic syndrome; NIDDM, noninsulin dependent diabetes mellitus; OB, obesity; OW, overweight; PCOS, polycystic ovarian syndrome.
- Sample size: *A*, analysed; *R*, randomised.
- Gender: M, male; F, female.
- Country: AU, Australia; CN, China; JP, Japan; KR, Korea.
- Duration: *d*, day; min, minutes; *m*, months; *w*, weeks.
- Intervention: CHM, Chinese herbal medicine; C, control group; LI, lifestyle intervention; PL, placebo; *T*, treatment group; WM, Western medication.

A total of 39 CHM formulas, including two repeats, were investigated. Twenty studies used traditional decoction [21, 22, 25, 27, 29–31, 35, 37, 39, 40, 43–49, 51, 55], eight studies used capsules [20, 24, 26, 32–34, 36, 38], seven studies used granules [28, 41, 42, 50, 52, 56, 57], three studies used tablets [19, 23, 53], and one study used powder [53]. Details of CHM formulas with herbal ingredients are listed in Table S2. The two most frequently used formulas were Crataegi Fructus lipid-lowering capsule (Shan Zha Xiao Zhi Jiao Nang) and a formula of Eupatorii Herba, Ephedrae Herba, and Coptidis Rhizoma (Pei Lian Ma Huang Fang). The top ten most commonly used herbs were Poria Cocos (Fu Ling) (*n* = 12), Coptidis Rhizoma (Huang Lian) (*n* = 12), Crataegi Fructus (Shan Zha) (*n* = 12), Glycyrrhizae Radix (Gan Cao) (*n* = 11), Nelumbinis Folium (He Ye) (*n* = 11), Pinelliae Rhizoma (Ban Xia) (*n* = 10), Atractylodis Macrocephalae Rhizoma (Bai Zhu) (*n* = 9), Citri Reticulatae Pericarpium (Chen Pi) (*n* = 9), Alismatis Rhizoma (Ze Xie) (*n* = 9), and Atractylodis Rhizoma (Cang Zhu) (*n* = 8).

### 3.2. Risk of Bias Assessment

**Figure 2:**
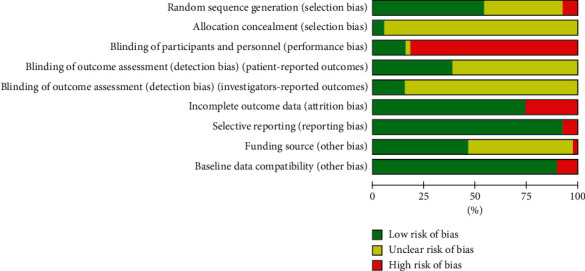
Summary of risk of bias for each domain among the 39 included studies.

**Figure 3:**
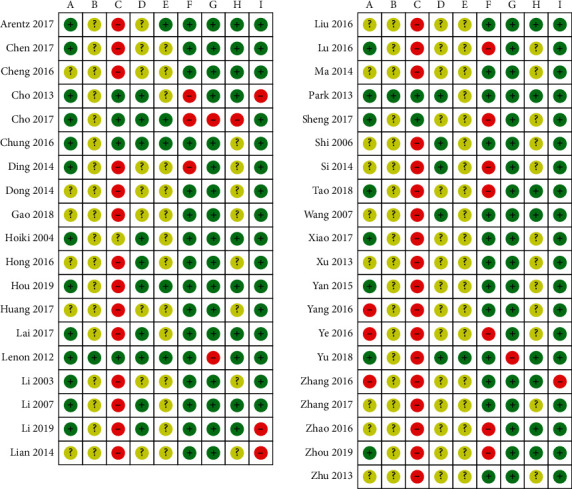
Risk of bias graph: review authors' judgements about each risk of bias item presented as percentages across all included studies. (a) Random sequence generation (selection bias). (b) Allocation concealment (selection bias). (c) Blinding of participants and personnel (performance bias). (d) Blinding of outcome assessment (detection bias) (patient-reported outcomes). (e) Blinding of outcome assessment (detection bias) (investigators-reported outcomes). (f) Incomplete outcome data (attrition bias). (g) Selective reporting (reporting bias). (h) Funding source (other bias). (i) Baseline data compatibility (other bias).

Twenty studies reported adequate methods of random sequence generation, including computer software [19, 22–24, 33, 41, 52] and random number table [20, 21, 25, 28, 30, 35, 36, 39, 42, 45, 47, 49, 56]. Sequence allocation was only concealed in two studies [33, 41], while blinding of participants and personnel was performed in six studies [22–24, 33, 41, 42]. Most studies were assessed as a high risk of bias for blinding of participants and personnel because the treatment and control groups received different forms of intervention. The majority of studies (*n* = 24) did not mention how body weight or BMI was measured and hence they were rated as unclear risk of patient-reported bias. The remaining studies (*n* = 15) were assessed as low risk of patient-reported bias because they either described the intervals and location in which outcome measures were assessed or indicated specific methods of measurement such as the placement of measuring tape or the accuracy of measurement records. The majority of included studies did not report whether they blinded their outcome assessors hence only six studies [19, 23, 24, 30, 33, 52], who specified the independent assessment of outcome assessors or the blinding of investigators, were reported as low risk of bias for this domain. Nine studies [23, 25, 39, 42, 44, 45, 51, 55, 56] excluded noncompliers and performed per-protocol analysis, while the rest of the studies either reported their outcome data with intention-to-treat analysis or did not subject to any dropouts. The risk of selective reporting bias for four articles [23, 33, 41, 52] was assessed based on published protocol, while that of remaining studies was compared against their published reports only. Three of four studies [23, 33, 52] reported slightly different outcome measures in their results section as compared to their registered protocol. Only a small difference was detected in these three studies: Cho et al. [23] had an addition of serum lipid profile outcomes in the published article; Lenon et al. [33] employed resting metabolic rate outcomes in the trial, and Yu et al. [52] published several primary and secondary efficacy outcomes including insulin resistance index (HOMA-IR), *β*-cell function index (HOMA-*β*), and BMI. The remaining studies reported all outcome measures mentioned in the methods section and were assessed as low risk of reporting bias. In terms of funding source and conflicts of interest, 18 studies [19–22, 28, 30, 32, 33, 35, 36, 38, 41, 45, 46, 52, 53, 55, 56] stated that they were supported by not-for-profit institutions, such as the national scientific funding or local scientific grant. One study [23] was funded by the pharmaceutical company which supplied medication for the intervention group, which could lead to potential conflicts of interest. The remaining 20 did not specify their funding sources and hence were difficult to determine potential competing interests. Baseline data in four studies were incomparable when assessed using RevMan 5.4.1 and thus those four studies were excluded from the meta-analyses [22, 35, 37, 53]. The risk of bias of 39 included studies is summarised in Figures 2 and 3.

### 3.3. Clinical Effects

#### 3.3.1. Body Weight

**Figure 4:**
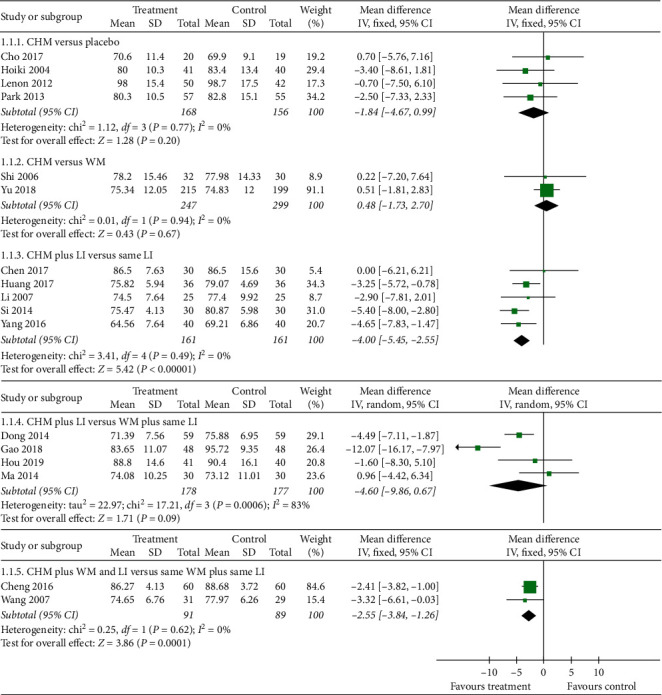
Comparison of body weight between Chinese herbal medicine treatment and control groups.

Twenty-two studies reported body weight as an outcome measure. However, three of them [22, 35, 53] did not have comparable baseline, and two lacked data [19, 24]. The pooled data from the remaining 17 [20, 21, 23, 26–28, 30, 31, 33, 36, 40, 41, 43, 44, 46, 50, 52] showed no significant difference between CHM and placebo (MD −1.84, 95% CI −4.67 to 0.99, *I*^2^ = 0%; *n* = 4) [23, 28, 33, 41] or between CHM and WM (MD 0.48, 95% CI −1.73 to 2.70, *I*^2^ = 0%; *n* = 2) [43, 52]. However, there was a statistically significant effect favouring CHM when CHM is combined with LI compared to same LI (MD −4.00, 95% CI −5.45 to −2.55, *I*^2^ = 0%; *n* = 5) [20, 31, 36, 44, 50]. In the comparison of CHM plus LI versus WM plus same LI, no significant difference was observed (MD −4.60, 95% CI −9.86 to 0.67, *I*^2^ = 83%; *n* = 4) [26, 27, 30, 40]. Finally, when CHM was used as an adjunct to WM and LI and compared to same WM and LI, a significant difference was revealed (MD −2.55, 95% CI −3.84 to −1.26, *I*^2^ = 0%; *n* = 2) [21, 46]. Forest plot of body weight comparing treatment and control groups is illustrated in Figure 4.

#### 3.3.2. BMI

**Figure 5:**
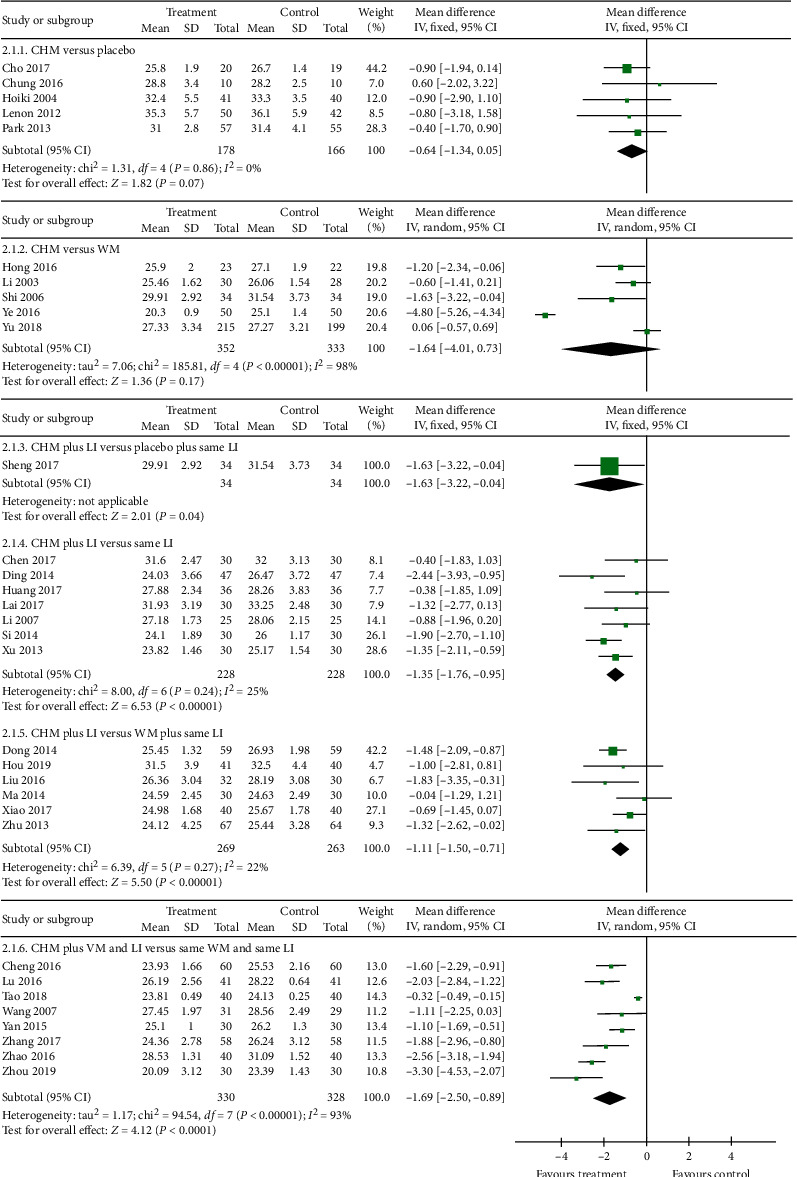
Comparison of body mass index between Chinese herbal medicine treatment and control groups.

Thirty-seven studies reported BMI at baseline and the end of treatment. However, the baseline data of four studies [22, 35, 37, 53] were incomparable and one study [19] did not report sufficient data. Thus, these were excluded from the meta-analysis. The pooled results revealed that there was no significant difference in BMI between CHM and placebo (MD −0.64, 95% CI −1.34 to 0.05, *I*^2^ = 0%; *n* = 5) [23, 24, 28, 33, 41] or between CHM and WM (MD −1.64, 95% CI −4.01 to 0.73, *I*^2^ = 98%; *n* = 5) [29, 34, 43, 51, 52] at the end of treatment. One study comparing CHM plus LI with placebo plus same LI reported a significant favour of the CHM plus LI arm (MD −1.63, 95% CI −3.22 to −0.04; *n* = 1) [42]. Similarly, seven studies comparing CHM plus LI with same LI also yielded significant difference favouring CHM plus LI arm (MD −1.35, 95% CI −1.76 to −0.95, *I*^2^ = 25%; *n* = 7) [20, 25, 31, 32, 36, 44, 48]. Further, a significant effect was also detected in studies comparing CHM plus LI versus WM plus same LI (MD −1.11, 95% CI −1.50 to −0.71, *I*^2^ = 22%; *n* = 6) [26, 30, 38, 40, 47, 57]. Finally, studies using CHM combined with WM and LI as compared to same WM and LI reported a modest but significant favour of the treatment intervention (MD −1.69, 95% CI −2.50 to −0.89, *I*^2^ = 93%; *n* = 8) [21, 39, 45, 46, 49, 54–56]. The forest plot of BMI comparing treatment and control groups is illustrated in Figure 5.

### 3.4. Adverse Events

Adverse events were monitored in 16 studies [19, 21–24, 26–28, 33, 36, 40, 51, 52, 54, 56, 57]. Nine studies found no adverse reactions during the trial [20, 31, 37, 41, 42, 47–50]. The rest of the studies did not state whether safety assessments were investigated or adverse reactions were observed. There was no significant difference in the frequency of adverse events between CHM and placebo (RR 3.08, 95% CI 0.42 to 22.74, *I*^*2*^ = 84%; *n* = 4) [23, 24, 28, 33], CHM plus LI versus same LI (RR 6.11, 95% CI 0.75 to 49.48, *I*^*2*^ = 0%; *n* = 2) [19, 36], and CHM plus WM and LI versus same WM and LI (RR 1.67, 95% CI 0.62 to 4.47, *I*^*2*^ = 27%; *n* = 3) [21, 54, 56]. However, when CHM was combined with LI compared with WM and same LI, reduced risk of adverse events was observed in the treatment arm (RR 0.20, 95% CI 0.11 to 0.37, *I*^*2*^ = 7%; *n* = 4) [26, 27, 40, 57].

The most frequent types of adverse events in CHM groups were gastrointestinal conditions including abdominal discomfort or distension, indigestion, nausea, vomiting, diarrhoea, while the most common adverse events in the control groups were abdominal flatus, steatorrhoea, oily stools, and diarrhoea. One study [28] reported the increased frequency of diarrhoea as a result of Natrium Sulphuricum and Rhei Rhizoma, and another study [26] reported six cases of diarrhoea associated with the intake of metformin. The rest of the studies did not attribute specific medications to reported adverse events.

### 3.5. Subgroup and Sensitivity Analyses

Subgroup meta-analyses were planned for the treatment period and the form of CHM. However, due to the limited number of included studies in each comparison, they could not be performed.

For body weight outcomes, a high heterogeneity (83%) was present in the comparison of CHM plus LI versus WM plus same LI. An outlying study was identified [27]; it reported a mean weight loss of 16 kg at the end of 12-week treatment. In comparison to Hou et al. 2019 [30], a study with similar sample size, study design, and interventions, only an average of 1.60 kg weight loss was achieved, nevertheless results from [30] were not significant due to a relatively large variance from −8.30 kg to 5.10 kg. Upon excluding the outlying study [27], heterogeneity was reduced from 83% to 42%, and a significant difference emerged between the CHM plus LI versus WM plus same LI groups (MD −3.24, 95% CI −5.47 to −1.02, *I*^*2*^ = 42%; *n* = 3) (Figure S1).

For BMI outcomes, a high heterogeneity (*I*^*2*^ = 98%) in the CHM versus WM group was observed. In this comparison, four out of five studies applied metformin [29, 34, 43, 52], while another study used lipid-lowering agents (atorvastatin) [51]. By removing the study using atorvastatin [51] from the meta-analysis, heterogeneity reduced from 98% to 53% and yet a significant BMI reduction in the CHM treatment group was not achieved (MD −0.64, 95% CI −1.34, 0.06, *I*^2^ = 53%; *n* = 4) (Figure S2). Similarly, no significant difference was found on body weight outcome within this subgroup (CHM versus WM), indicating CHM was not superior over WM.

### 3.6. Publication Bias

**Figure 6:**
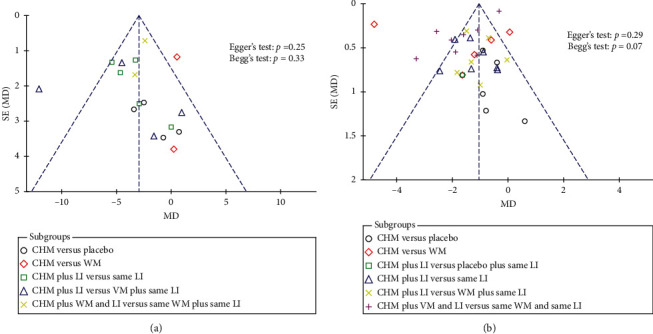
Funnel plots for overall (a) body weight (kg), and (b) body mass index (kg/m^2^) of included studies based on subgroups, represented within 95% confidence limits. CHM, Chinese herbal medicine; LI, lifestyle intervention; MD, mean difference; SE, standard error; WM, Western medication.

The visual inspection of funnel plots for end-of-treatment body weight and BMI outcomes revealed asymmetry, suggesting a risk of publication bias in overall included studies favouring the CHM intervention group compared to its respective control (Figure 6). However, quantitative analyses of small study effects did not reveal evidence of significant publication bias for body weight (Egger's test: *p*=0.25; Begg's test: *p*=0.33) or BMI outcomes (Egger's test: *p*=0.29; Begg's test: *p*=0.07).

## 4. Discussion

This review evaluated the effects of CHM against placebo, LI, and WM, with or without cointerventions, on the end-of-treatment body weight and BMI outcomes among 3415 overweight/obese adult participants. No significant therapeutic effect was found when CHM was administered as a single therapy against placebo (no active ingredients) or WM (metformin, atorvastatin) on both outcomes, or as a cointervention against WM (metformin or orlistat) on body weight outcome. When CHM was added on to LI compared to the same LI, and to WM plus LI compared to the same WM plus LI, significantly lower body weight and BMI were achieved. Similarly, when CHM was coadministered with LI, they yielded substantially lower BMI compared to placebo or WM with the same LI cointerventions.

After performing sensitivity analyses and excluding individual studies with high population or methodological confounding factors, a trend favouring CHM as adjunctive therapies or cointerventions consistently emerged on both outcomes. A significantly lower end-of-treatment mean difference on body weight and BMI outcomes was demonstrated when CHM is administered as an adjunct to LI (−4.00 kg, −1.35 kg/m^2^) and WM plus LI (−2.55 kg, −1.69 kg/m^2^). When CHM is coadministered with LI, a lower body weight compared to WM (−3.24 kg) and lower BMI compared to placebo (−1.63 kg/m^2^) and WM (−1.11 kg/m^2^) were achieved. Single therapy of CHM versus placebo or WM on body weight and BMI outcomes remained insignificant. Sensitivity analyses suggest that the pooled effects of included studies within the intervention subgroups were not sufficiently robust, hence caution is needed when interpreting the results. Nevertheless, this finding has echoed the recommendations specified in N57 Obesity Clinical Practice Guidelines as “multicomponent interventions that are delivered through multidisciplinary care may be more effective than interventions delivered by individual health professionals” [4].

The quality of the included studies varied in different domains; this is consistent with findings from other reviews [11, 12]. While all studies claimed that they were randomised, not all provided methods on randomisation and allocation concealment claimed. The lack of reporting or implementation of appropriate randomisation may have introduced selection bias in the interest of the treatment group. Given the difficulties inherent in masking Chinese herbal medicines due to its odour, colour, and taste, the standardisation of interventional form (tablet, capsule, or pill) within two intervention groups may prevent differential care, improve blinding of outcome assessors, and facilitate with adherence. Future clinical trials may consider adopting a matching placebo to minimise potential performance and detection biases.

Findings from this review concerning the reporting of trials were supported by two existing systematic reviews, one [12] searched up to July 2009 and the other [11] up to February 2010. With the introduction of the CONSORT statement in 2010, the quality of RCTs in other health professions has increased dramatically [58–60]. However, our review did not note the trend of quality improvement over the years. It may be due to the delay in translation and dissemination of the CONSORT statement to non-English speaking population. It is recommended that future studies report findings adhering to the CONSORT statement to enable sufficient data for synthesis.

Possible pathways for CHMs to alleviate obesity conditions are hunger suppression, metabolic regulation, insulin sensitivity enhancement, and energy expenditure modulation [61, 62]. For instance, a comprehensively studied formulation (Bofu-Tsusho-San) demonstrated antiobesity effects by increasing thermogenesis of brown adipose tissue and inhibiting phosphodiesterase activities in MSG-obese mice models [63], reducing triglycerides, glucose, insulin, and leptin levels in high fat diet-induced mice after a 25-day treatment [64] and preventing adipogenesis via gene expression modulation reflected in microassay profiling studies [65, 66]. Empirical evidence of Bofu-Tsusho-San has also revealed significant effects on lowering body weight, reducing levels of low-density lipoprotein cholesterol, triglycerides, and blood glucose with considerable tolerability [28] and alleviating obesity-related hypertension [67] in both early and later phases of obesity [68].

Our review has identified 10 commonly used CHMs for weight management. Seven of ten are consistent with findings from [11], including Crataegi Fructus (Shan Zha), Atractylodis Macrocephalae Rhizoma (Bai Zhu), Alismatis Rhizoma (Ze Xie), Poria (Fu Ling), Nelumbinis Folium (He Ye), Atractylodis Rhizoma (Cang Zhu), and Citri Reticulatae Pericarpium (Chen Pi). These CHMs have been widely used in Chinese medicine clinical practice for their actions to transform dampness and clear heat and regulate and strengthen the digestive system. In Chinese medicine, obesity is predisposed by two different bodily phenotypes: (1) excessive consumption of high energy nutrition causing accumulation of phlegm, damp, and heat in the body, and (2) weakness of the digestive system leading to inefficient metabolism [69]. Thus, the abovementioned CHMs are appropriate for reducing weight. More studies on their mechanisms of actions and associated signalling pathways are recommended.

Given the complex bodily interactions between the nervous and hormonal feedback systems that are responsible for homeostasis and thermogenesis, multireceptor targets as utilised in Chinese herbal formulations, coupled with lifestyle interventions, may be necessary for noninvasive yet holistic management of overweight and obesity. The approach of combining interdisciplinary modalities has been highlighted in guidelines for primary care in countries including Australia [4], United Kingdom [70], United States of America [71], Canada [72], and across Europe [73].

## 5. Conclusions

CHM could improve body weight and BMI in overweight and obese individuals when used as an adjunct therapy to LI with or without WM. However, due to a variety of Chinese herbal formulas used in the included studies, further studies focusing on the effects of individual formulas for weight management and their mechanisms of actions are required. In addition, a multidisciplinary approach involving CHM, LI, and/or WM is highly recommended as the intervention of choice to offer the best chance of effective weight management in a rigorously designed, large-scale RCT.

## Figures and Tables

**Table 1:** Characteristics of 39 included studies.

Study ID	Characteristics	Country	Sample size (R/A)	Gender, *T* (M : F)/C (M : F)	Age mean (SD)	Baseline body weight, *T*/C mean (SD)	Baseline BMI, *T*/C mean (SD)	Duration	Treatment group	Control group
*CHM versus placebo*										
Cho 2013^*∗*^ [22]	OW	KR	39 : 30/30 : 23	3 : 27/5 : 18	42.90 (12.67)/41.83 (14.82)	71.84 (10.57)/67.89 (7.85)	28.35 (3.95)/26.51 (2.21)	2 m	CHM: target herbal ingredient, 50 mL, tid	Placebo: THI (no herbal ingredient), 50 mL, tid
Cho 2017 [23]	OW	KR	30 : 30/20 : 19	10 : 20/8 : 22	39.5 (11.2)/41.7 (11.1)	75.0 (11.0)/72.6 (10.8)	27.2 (1.5)/27.1 (1.2)	12 w	CHM: YY-312, 3 tablets 400 mg each, bid	Placebo: placebo (no herbal ingredient), 3 tablets 400 mg each, bid
Chung 2016 [24]	OW, COB, MET	KR	13 : 13/10 : 10	6 : 4/6 : 4	50.00 (5.85)/45.20 (9.52)	79.30 (14.16)/80.96 (11.16)	29.5 (3.6)/28.9 3.0)	8 w	CHM: Qingxue Dan, 3 capsules 300 mg each, qd	Placebo: placebo (no herbal ingredient), 3 capsules 300 mg each, qd
Hioki 2004 [28]	OB, IGT	JP	44 : 41/41 : 40	0 : 41/0 : 40	52.6 (14.0)/54.8 (12.5)	90.8 (17.9)/90.3 (12.2)	36.7 (6.8)/36.1 (3.3)	24 w	CHM: Bofu-Tsusho-San (ephedrine 24 mg/day and inhibition of cAMP phosphodiesterase activity corresponding to 280 mg caffeine/day, 24 mg/day, tid	Placebo: placebo, 24 mg/day, tid
Lenon 2012 [33]	OB	AU	59 : 58/50 : 42	10 : 49/10 : 48	39.3 (13.2)/40.4 (10.2)	99.5 (15.1)/98.2 (17.3)	35.9 (4.9)/35.9 (5.9)	12 w	CHM: RCM-104, 4 capsules 500 mg each, tid	Placebo: placebo (no herbal ingredient), 4 capsules 500 mg each, tid
Park 2013 [41]	OB, HBP, NIDDM, HLD	KR	58 : 55/57 : 55	7 : 50/10 : 45	39.2 (9.5)/38.8 (10.1)	82.2 (10.5)/83.7 (14.4)	31.8 (2.60)/31.9 (3.80)	12 w	CHM: TJ001 extract, 7 g, tid	Placebo: placebo extract, 7 g, tid

*CHM versus WM*										
Hong 2016 [29]	OW, OB, PCOS	CN	23 : 22/23 : 22	0 : 23/0 : 22	24.3 (5.8)/25.1 (6.2)	NR	27.9 (2.6)/28.3 (2.8)	3 m	CHM: Jian Pi Qu Tan Tong Luo Fang, 200 mL/day, bid	WM: metformin tablet, 500 mg, tid
Li 2003 [34]	OW, OB, NIDDM, HLD	CN	30 : 28/30 : 28	NR	NR	NR	28.23 (1.40)/27.87 (1.36)	8 w	CHM: An Yi Jiao Nang, 3 capsules 0.35 g each, tid, 3.15 g/day	WM: metformin tablet, 0.5 g, tid
Shi 2006 [43]	OW, OB, IGT	CN	32 : 30/32 : 30	17 : 15/15 : 15	60-76yo/60-78yo	82.34 (11.18)/82.16 (12.23)	NR	8 w	CHM: Fufang Cangzhu Tang, 150 mL/day, bid	WM: metformin, 0.25 g, tid
Ye 2016 [51]	COB, HLD	CN	50 : 50/50 : 50	27 : 23/28 : 22	42.7 (9.5)/42.9 (10.1)	NR	27.1 (2.8)/27.4 (2.8)	1 m	CHM: Qu Tan Tiao Zhi Tang, 500 mL/day, bid	WM: atorvastatin tablet, 10 mg, qd
Yu 2018 [52]	OB, NIDDM	CN	225 : 225/215 : 199	104 : 111/98 : 101	52.82 (9.01)/52.90 (8.52)	77.82 (12.08)/76.86 (12.06)	28.24 (3.31)/28.01 (3.22)	12 w	CHM: Jiang Tang Tiao Zhi granule, 1 bag, bid	WM: metformin tablet, 0.25 g tid

*CHM plus LI versus same LI*										
Arentz 2017^*∗*^ [19]	OW, OB, PCOS	AU	60 : 62/60 : 62	0 : 60/0 : 62	29.2 (5.6)/28.9 (5.6)	93.2 (18.9)/97.3 (21.3)	34.1 (7.2)/35.2 (6.8)	3 m	CHM: Tablet 1, 3 tablets, qd; MediHerb Tribulus Forte, 3 tablets, qd. for 10 day LI: same as comparator	LI: diet follows evidence-based guidelines, exercise for at least 150 min per week including 90 min of aerobic activity
Chen 2017 [20]	OB, NAFLD	CN	30 : 30/30 : 30	19 : 11/17 : 13	32.8 (7.97)/37.4 (11.5)	88.4 (8.70)/87.2 (15.5)	32.4 (2.32)/32.3 (3.03)	12 w	CHM: Shan Zha Xiao Zhi Jiao Nang, 3 capsules, tid LI: same as comparator	LI: diet control, exercise routine
Ding 2014 [25]	OW, OB	CN	47 : 47/47 : 47	18 : 29/16 : 31	37.3 (9.8)/36.7 (9.6)	NR	28.92 (3.91)/28.71 (3.86)	4 w	CHM: Jian Pi Hua Zhuo Tang, 400 mL/day, bid LI: Same as comparator	LI: low fat, low sugar diet with 60 min exercise for 5 times weekly
Huang 2017 [31]	OB	CN	36 : 36/36 : 36	19 : 17/20 : 16	43.3 (16.21)/42.1 (17.42)	81.52 (6.31)/82.16 (5.67)	31.14 (3.57)/30.51 (3.09)	8 w	CHM: Jia Wei Ling Gui Zhu Gan Tang, 150 mL/day, tid LI: same as comparator	LI: diet control and 30 min aerobic exercise for 5 times weekly
Lai 2017 [32]	OB	CN	30 : 30/30 : 30	14 : 16/14 : 16	32.13 (8.18)/34.16 (9.46)	NR	32.62 (3.34)/33.16 (2.33)	12 w	CHM: Shan Zha Xiao Zhi Jiao Nang, 1 capsule 0.7 g, tid LI: same as comparator	LI: according to the “Chinese Adult Obesity Prevention and Control Guidelines”
Li 2007 [36]	OW, OB	CN	25 : 25/25 : 25	11 : 14/10 : 15	42.76 (9.79)/43.44 (14.02)	80.68 (8.35)/80.40 (9.62)	29.48 (1.91)/29.07 (2.15)	60 d	CHM: Jian Fei Tiao Zhi Jiao Nang, 4 capsules 0.5 g each, tid LI: same as comparator	LI: strict diet control, no alcohol, 30 min exercise for 3 times weekly
Li 2019^*∗*^ [35]	OW, OB	CN	27 : 28/27 : 28	11 : 16/13 : 15	39.86 (7.23)/31.17 (1.98)	87.16 (8.61)/92.43 (8.47)	32.41 (1.89)/31.17 (1.98)	12 w	CHM: Pei Lan Ma Huang Fang, 300 mL/day, bid LI: same as comparator	LI: dietary intake 1000–1500 kcal/day, 30–45 min aerobic exercise for 3–5 times weekly
Si 2014 [44]	OB	CN	30 : 30/30 : 30	47 : 13	36.25 (8.17)	82.33 (4.53)/81.83 (5.91)	29.94 (1.74)/29.40 (1.80)	2 m	CHM: Wen Shen Jian Pi Hua Tan Fang, 1 decoction/day, qd LI: same as comparator	LI: low calorie, high fibre diet, exercise routine
Xu 2013 [48]	OW, OB	CN	30 : 30/30 : 30	10 : 20/12 : 18	NR	73.24 (7.78)/74.87 (6.76)	NR	6 m	CHM: Dao Tan Tang Jia Jian, 200 mL/day, bidvLI: same as comparator	LI: diet control, exercise routine
Yang 2016 [50]	OW, OB	CN	40 : 40/40 : 40	21 : 19/18 : 22	45.63 (18.13)/46.51 (17.34)	NR	27.19 (1.36)/26.93 (1.21)	12 w	CHM: Qu Tan Qing Wei Fang granule, bidvLI: same as comparator	LI: reduce sugary/oily foods, no smoking or drinking, 30 min aerobic exercise 3 times weekly
Zhang 2016^*∗*^ [53]	OW, OB	CN	42 : 42/42 : 42	8 : 34/10 : 32	30 (12)/30 (9)	79.0 (10.20)/78.9 (10.70)	24.43 (3.39)/28.90 (3.32)	60 d	CHM: Jin Long Jiang Zhi San, 20 g, tidvLI: same as comparator	LI: low carbohydrate, low fat, low sugar diet, 80% full meals, water intake 5 mL/day

*CHM plus LI versus placebo plus same LI*										
Sheng 2017 [42]	OB	CN	35 : 35/34 : 34	10 : 24/13 : 21	37.74 (12.39)/39.29 (10.11)	NR	31.68 (2.87)/31.77 (4.07)	28 d	CHM: Jian Pi Shu Gan Jiang Zhi Fang, granule, bid LI: same as comparator	PL: placebo granule, bidvLI: low sugar, sodium, fat, high protein diet, no binge eating, sufficient aerobic exercise, increase fat-burning exercise

*CHM plus LI versus WM plus same LI*										
Dong 2014 [26]	COB, MET	CN	61 : 61/59 : 59	33 : 26/32 : 27	42.7 (4.6)/43. (4.7)	76.35 (7.56)/75.88 (6.95)	28.31 (1.29)/28.71 (1.23)	120 d	CHM: Hong He Qing Jiang capsule, 4 capsules, tid LI: same as comparator	WM: metformin 0.5 g bid; captopril 25 mg bid; simvastatin 10 mg qd LI: health education, diet control, increase physical activity
Gao 2018 [27]	OW, OB	CN	48 : 48/48 : 48	28 : 20/25 : 23	42.3 (11.6)/40.2 (13.1)	99.58 (8.43)/100.31 (10.25)	NR	12 w	CHM: Hua Tan Qu Yu Jian Fei Tang, 100 mL/day, bid LI: same as comparator	WM: orlistat, one tablet, tid LI: abstain from strong flavour and difficult-to-digest foods, reduce carbohydrates, increase fruits, vegetables, and exercise
Hou 2019 [30]	OB	CN	41 : 40/41 : 40	NR	18–65	91.4 (14.7)/92.1 (16.9)	32.4 (4.0)/33.3 (4.6)	12 w	CHM: Xie Re Hua Zhuo Fang, 1 decoction/day, bid LI: same as comparator	WM: orlistat tablet, 120 mg, bid LI: calorie restrict 1500–1800 kcal/day; exercise 40–60 min for 5 times weekly

Liu 2016 [38]	OW, OB, NAFLD	CN	32 : 30/32 : 30	18 : 14/16 : 14	39.5 (10.2)/39.1 (9.1)	NR	29.82 (3.35)/29.06 (3.15)	6 m	CHM: Qiang Gan Jiao Nang, 1 capsule 2.0 g, bid LI: same as comparator	WM: atorvastatin tablet, 20 mg, qd LI: no alcohol, reduce calorie intake, increase exercise
Ma 2014 [40]	OW, OB	CN	30 : 30/25 : 25	NR	NR	78.11 (10.08)/78.16 (9.88)	28.61 (2.78)/28.72 (2.14)	3 m	CHM: Pei Lian Ma Huang Fang, 150 mL/day, bid LI: same as comparator	M: orlistat tablet, 0.12 g, tid LI: calorie intake 1000–1500 kcal/day; aerobic activity 30 min, 3–5 times weekly
Xiao 2017 [47]	OB, IGT	CN	40 : 40/40 : 40	24 : 16/26 : 14	52.8 (7.8)/53.2 (6.5)	NR	26.15 (2.13)/26.14 (2.26)	3 m	CHM: Jia Wei Xiao Xian Xiong Tang, 300 mL/day, bid LI: same as comparator	WM: acarbose, 50 mg, tid LI: diet control, exercise routine
Zhu 2013 [57]	OB, IGT	CN	74 : 70/67 : 64	53 : 21/47 : 23	46.3 (4.6)/48.6 (3.2)	NR	30.57 (3.24)/30.70 (3.11)	6 m	CHM: Sheng Yang Li Shi Fang Ke Li Chong Ji, 100 mL, bid LI: same as comparator	WM: metformin, 0.5 g, tid LI: adjust ratio of 3 major nutrients, exercise 30 min/day

*CHM plus WM and LI versus same WM and LI*										
Cheng 2016 [21]	OW, OW, NIDDM	CN	60 : 60/60 : 60	NR	NR	92.00 (4.48)/90.30 (3.49)	26.67 (1.99)/26.52 (2.17)	12 w	CHM: Fu Fang Fan Shi Liu Zhi Ji, 100 mL/day, tid WM and LI: same as comparator	WM: metformin tablet, 0.25 g, tid LI: diet control and exercise routine
Lian 2014^*∗*^ [37]	OB, IR	CN	30 : 30/30 : 30	11 : 16/13 : 15	39.00 (18.25)/41.00 (17.50)	NR	23.72 (4.71)/28.23 (2.73)	12 w	CHM: Fei Pang No.1 formula, 200 mL/day, bid WM and LI: same as comparator	WM: metformin tablet, 850 mg, bid LI: basic calorie 3347–6276 kJ/day, balance 3 major nutrients, reduce sweet/oily food, no alcohol, exercise 30 min for 3 times weekly
Lu 2016 [39]	OB, IR	CN	41 : 41/41 : 41	24 : 17/22 : 19	38.8 (5.7)/39.5 (5.6)	NR	29.82 (2.62)/30.09 (2.58)	24 w	CHM: Cang Chai Tiao Zhong Tang, 1 decoction/day, bid WM and LI: same as comparator	WM: sitagliptin tablet, 100 mg, qd LI: diet regulation, avoid high sugar and fat foods, no smoking, alcohol, or snacks, exercise 30 min for 2–4 times weekly
Tao 2018 [45]	OW, OB, NIDDM	CN	40 : 40/40 : 40	21 : 19/23 : 17	49.15 (11.29)/49.78 (11.09)	NR	25.20 (0.67)/24.98 (0.31)	12 w	CHM: Jian Pi Qu Shi Fang, 200 mL/day, bid WM and LI: same as comparator	WM: metformin tablet, 0.5 g, tid LI: diet control, sufficient exercise
Wang 2007 [46]	OW, OB, HBP	CN	31 : 29/31 : 29	19 : 12/18 : 11	50.97 (11.10)/49.24 (10.07)	76.18 (6.88)/78.41 (6.44)	28.02 (2.17)/28.72 (2.23)	8 w	CHM: Ping Gan Yi Shen Tiao Tan Yin, 1 decoction/day, bid WM and LI: same as comparator	WM: benazepril, 10 mg, qd LI: sufficient exercise and reasonable diet
Yan 2015 [49]	OW, OB, MET	CN	30 : 30/30 : 30	17 : 13/19 : 11	33.7 (7.56)/32.9 (7.17)	NR	28.1 (1.1)/27.9 (1.2)	8 w	CHM: Wu Ling San Jia Wei, 1 bag, bid WM and LI: same as comparator	WM: metformin tablet, 0.5 g, tid LI: heath education, aerobic exercise
Zhang 2017 [54]	OB, NIDDM	CN	58 : 58/58 : 58	38 : 20/37 : 21	46.04 (10.19)/46.30 (10.49)	NR	29.48 (3.48)/29.18 (3.59)	60 d	CHM: Tian Mai Xiao Ke Pian, 2 tablets, bid WM and LI: same as comparator	WM: sitagliptin 1 tablet, qd LI: strict diet control
Zhao 2016 [55]	OB, HBP	CN	40 : 40/40 : 40	11 : 29/18 : 22	62.34 (9.32)/64.18 (8.67)	NR	31.04 (1.33)/30.88 (1.79)	12 w	CHM: Ban Xia Bai Zhu Tian Ma Tang, 400 mL/day, bid WM and LI: same as comparator	WM: valsartan tablet 80 mg, qd LI: low sodium, low fat diet
Zhou 2019 [56]	OW, PCOS	CN	30 : 30/30 : 30	0 : 30/0 : 30	27.20 (3.73)/27.80 (4.35)	NR	26.88 (2.20)/26.76 (2.03)	3 m	CHM: He Qi San, 1 sachet, bid WM and LI: same as comparator	WM: metformin, 500 mg, qd LI: increase exercise, reduce oily, sugary, and raw foods

Notes: ^*∗*^Not included in meta-analysis; NR, not reported.

## Data Availability

The data are available upon request to the corresponding author.

## Abbreviations

BMI: Body mass index
CHM: Chinese herbal medicine
CI: Confidence interval
CONSORT: Consolidated standards of reporting trials
FDA: Food and drug administration
LI: Lifestyle intervention
MD: Mean difference
PRISMA: Preferred reporting items for systematic reviews and meta-analysis
RCT: Randomised controlled trial
RR: Risk ratio
WHO: World Health Organisation
WM: Western medication.
CHM: Chinese herbal medicine
LI: Lifestyle intervention
WM: Western medication.
CHM: Chinese herbal medicine
LI: Lifestyle intervention
WM: Western medication.

## Supplementary Materials

Supplementary Materials The supplementary file includes the following tables and figures: Table S1: search strategy example from PubChem used to identify studies related to Chinese herbal medicine for weight management. Table S2: details of Chinese herbal treatment in included studies. Figure S1: forest plot demonstrating the effects of CHM plus LI versus WM plus same LI on body weight after excluding outlying study. Figure S2: forest plot demonstrating the effects of CHM versus WM on body mass index after excluding outlying study.
