# Supplementary material for: Chinese Herbal Medicine for Weight Management: A Systematic Review and Meta-Analyses of Randomised Controlled Trials
Source: J Obes. 2021 Mar 9;2021:3250723. doi: 10.1155/2021/3250723 (PMC7964117; doi:10.1155/2021/3250723)
Supplement: Supplementary Materials — The supplementary file includes the following tables and figures: Table S1: search strategy example from PubChem used to identify studies related to Chinese herbal medicine for weight management. Table S2: details of Chinese herbal treatment in included studies. Figure S1: forest plot demonstrating the effects of CHM plus LI versus WM plus same LI on body weight after excluding outlying study. Figure S2: forest plot demonstrating the effects of CHM versus WM on body mass index after excluding outlying study. [file 3250723.f1.docx]

# Supplementary files

**Table S1: PubMed search strategy example**

| PubMed search strategy example^¶^ |
| --- |
| #1: Obesity [MeSH]  #2: Anti-obesity [MeSH]  #3: Overweight [MeSH]  #4: Body Mass Index [MeSH]  #5: Weight*  #6: #1 OR #2 OR #3 OR #4 OR #5  #7: Drugs, Chinese Herbal [MeSH]  #8: Traditional Chinese Medicine  #9: Plant extracts [MeSH]  #10: Herb*  #11: Korean medicine  #12: Kampo medicine  #13: #7 OR #8 OR #9 OR #10 OR #11 OR #12  #14: Random*  #15: Placebo  #16: Double-blind  #17: Clinic*  #18: Trial*  #19: #14 OR #15 OR #16 OR #17 OR #18  #20: #6 AND #13 AND #19 |

^¶^Title and keywords of studies were searched using the above terms, they were modified to suit the indexing system on each database.

**Table S2: Details of Chinese herbal treatment in included studies**

| Chinese herbal formula | Ingredient |
| --- | --- |
| MediHerb Tribulus Forte | Glycyrrhizae Radix (Gan cao (zhi), Paeoniae Radix Alba (Bai shao), Cinnamomi Cortex (Rou gui), Hyperici perforati herba (Guan ye jin si tao), Tribuli Fructus (Ji li) |
| An Yi Jiao Nang | Astragali Radix (Huang qi), Rehmanniae Radix (Sheng di), Coptidis Rhizoma (Huang lian), Mori Cortex (Sang bai pi), Aucklandiae Radix (Mu xiang (guang)), Salviae Miltiorrhizae Radix et Rhizoma (Dan shen) |
| Ban Xia Bai Zhu Tian Ma Tang | Pinelliae Rhizoma (Ban xia (jiang)), Gastrodiae Rhizoma (Tian ma), Poria cocos (Fu ling), Citri Exocarpium Rubrum (Ju hong), Atractylodis Macrocephalae Rhizoma (Bai zhu), Glycyrrhizae Radix (Gan cao), Zingiberis Rhizoma (﻿Sheng jiang), Jujubae Fructus (Da zao) |
| Bofu-tsusho-san | Scutellariae Radix (Huang qin), Glycyrrhizae Radix (Gan cao), ﻿ Jie geng (Platycodi Radix), Gypsum Fibrosum (﻿Shi gao), ﻿Atractylodis Rhizoma (Cang zhu), ﻿Rhei Rhizoma (Da huang), ﻿Schizonepetae Spica (Jing jie), ﻿Gardeniae Fructus (Zhi zi), ﻿Paeoniae Radix (Chi shao), ﻿Cnidium Rhizoma (Chuan xiong), ﻿Angelicae Radix (Dang gui), ﻿Menthae Herba (Bo he), Ledebouriellae Radix (﻿Fang feng), ﻿Ephedrae Herba (Ma huang), Forsythiae Fructus (Lian qiao), ﻿Zingiberis Rhizoma (Sheng jiang), Talcum (Hua shi), ﻿Natrium Sulphuricum (Mang xiao) |
| Cang Chai Tiao Zhong Tang | Atractylodis Rhizoma (Cang zhu), Bupleuri Radix (Chai hu), Astragali Radix (Huang qi), Spatholobi Caulis (Ji xue teng), Coptidis Rhizoma (Huang lian), Mume Fructus (Wu mei), Coicis Semen (Yi yi ren), Mantidis Oötheca (Sang piano xiao), Citri Reticulatae Pericarpium (Chen pi), Paeoniae Radix Alba (Bai shao), Bambusae Caulis in Taenias (Zhu ru), Trichosanthis Fructus (Gua lou), Magnoliae Officinalis Cortex (Hou po) |
| Modified Dao Tan Tang | Pinelliae Rhizoma (Ban xia), Arisaematis Rhizoma (Nan xing (zhi)), Zingiberis Rhizoma (Sheng jiang), Citri Exocarpium Rubrum (Ju hong), Aurantii Fructus (Zhi shi), Benincasae Exocarpium (Dong gua pi), Alismatis Rhizoma (Ze xie), Raphani Semen (Lai fu zi), Atractylodis Macrocephalae Rhizoma (Bai zhu), Poria cocos (Fu ling), Bambusae Caulis in Taenias (Zhu ru), Trichosanthis Pericarpium (Gua lou), Coptidis Rhizoma (Huang lian), Glycyrrhizae Radix (Gan cao) |
| Fei Pang 1 Hao Fang | Poria cocos (Fu ling), Citri Reticulatae Pericarpium (Chen pi), Mori Folium (Sang ye), Gynostemmae Pentaphylli herba (Jiao gu lan), Nelumbinis Folium (He ye), Alismatis Rhizoma (Ze xie), Salviae Miltiorrhizae Radix et Rhizoma (Dan shen), Benincasae Exocarpium (Dong gua pi) |
| Fu Fang Fan Shi Liu Zhi Ji | Psidii Guajavae Folium (Fan shi liu ye), Euonymi Ramulus (Gui jian yu), Fici Simplicissimae radix (Wu zhi mao tao) |
| Fufang Cang zhu (Atractylodis Rhizoma) Tang | Atractylodis Rhizoma (Cang zhu), Coicis Semen (Yi yi ren), Mori Fructus (Sang shen), Dioscoreae Rhizoma (Shan yao), Phellodendri Chinensis Cortex (Huang bai), Litchi Semen (Li zhi he), Pheretima (Di long) |
| He Qi San | Nelumbinis Folium (He ye), Astragali Radix (Huang qi), Cassiae Semen (Jue ming zi), Polygoni Multiflori Radix (He shou wu), Dioscoreae Rhizoma (Shan yao), Benincasae Exocarpium (Dong gua pi), Acori Tatarinowii Rhizoma (Shi chang pu) |
| Hua Tan Qu Yu Jian Fei Tang | Nelumbinis Folium (Da He ye), Polygoni Multiflori Radix Preparatum (Shou wu (zhi)), Crataegi Fructus (Shan zha (sheng)), Cassia Semen (Jue ming), Salviae Miltiorrhizae Radix et Rhizoma (Dan shen), Polygoni Cuspidati Rhizoma et Radix (Hu zhang), Polygonati Rhizoma (Huang jing), Pinelliae Rhizoma (Ban xia (jiang)), Alismatis Rhizoma (Ze xie), Glycyrrhizae Radix (Gan cao) |
| Jia Wei Ling Gui Zhu Gan Tang | Poria cocos (Fu ling), Cinnamomi Ramulus (Gui zhi), Atractylodis Macrocephalae Rhizoma (Bai zhu), Codonopsis Radix (Dang shen), Crataegi Fructus (Shan zha (sheng)), Pinelliae Rhizoma (Ban xia (fa)), Siegesbeckiae Herba (Xi xian cao), Carthami Flos (Hong hua), Chuanxiong Rhizoma (Chuan xiong), Polygoni Multiflori Radix Preparatum (Shou wu (zhi)), Glycyrrhizae Radix (Gan cao) |
| Jia Wei Xiao Xian Xiong Tang | Coptidis Rhizoma (Huang lian), Trichosanthis Fructus (Gua lou), Aurantii Fructus (Zhi shi), Poria cocos (Fu ling), Atractylodis Rhizoma (Cang zhu), Bambusae Caulis in Taenias (Zhu ru), Pinelliae Rhizoma (Ban xia (qing)), Mori Folium (Sang ye), Cassiae Semen (Jue ming zi) |
| Jian Fei Tiao Zhi Jiao Nang | Rhei Rhizoma (Da huang (sheng)), Polygoni Multiflori Radix (He shou wu), Scutellariae Radix (Huang qin), Alismatis Rhizoma (Ze xie), Nelumbinis Folium (He ye), Crataegi Fructus (Shan zha) |
| Jian Pi Hua Zhuo Tang | Poria cocos (Fu ling), Cinnamomi Ramulus (Gui zhi), Astragali Radix Huang qi), Atractylodis Macrocephalae Rhizoma (Bai zhu), Lycii Fructus (Gou qi), Crataegi Fructus (Shan zha (sheng)), Rhei Rhizoma (Da huang) |
| Jian Pi Qu Shi Fang | Astragali Radix (Huang qi), Coicis Semen (Yi yi ren), Codonopsis Radix (Dang shen), Poria cocos (Fu ling), Dioscoreae Rhizoma (Shan yao), Atractylodis Macrocephalae Rhizoma (Bai zhu), Citri Reticulatae Pericarpium (Chen pi), Glycyrrhizae Radix (Gan cao (zhi)) |
| Jian Pi Qu Tan Tong Luo Fang | Atractylodis Macrocephalae Rhizoma (Bai zhu), Codonopsis Radix (Dang shen), Poria cocos (Fu ling), Atractylodis Rhizoma (Cang zhu), Cyperi Rhizoma (Xiang fu), Epimedii Folium (Xian ling pi), Cinnamomi Ramulus (Gui zhi), Bupleuri Radix (Chai hu), Angelicae Sinensis Radix (Dang gui), Juglandis Semen (Tao ren), Rehmanniae Radix Praeparata (Du huang (shu)), Spatholobi Caulis (Ji xue teng), Citri Reticulatae Pericarpium (Chen pi) |
| Jian Pi Shu Gan Jiang Zhi Fang | Atractylodis Rhizoma (Cang zhu (chao)), Bupleuri Radix (Chai hu (chao)), Pinelliae Rhizoma (Ban xia (zhi)), Cyperi Rhizoma (Xiang fu (zhi)), Poria cocos (Fu ling), Alismatis Rhizoma (Ze xie), Cassiae Semen (Jue ming zi), Nelumbinis Folium (He ye) |
| Jiang Tang Tiao Zhi | Aloe (Lu hui), Coptidis Rhizoma (Huang lian), Anemarrhenae Rhizoma (Zhi mu), Setariae Fructus Germinatus (Hong gu), Momordica Charantia (Ku gua), Salviae Miltiorrhizae Radix et Rhizoma (Dan shen), Schisandrae Chinensis Fructus (Wu wei zi), Zingiberis Rhizoma (Gan jiang) |
| Jin Long Jiang Zhi San | Ipomoea cairica (Wu zhao jin long), Coptidis Rhizoma (Huang lian), Pinelliae Rhizoma (Ban xia (qing)), Trichosanthis Semen (Gua lou ren), Paeoniae Radix Rubra (Chi shao) |
| Pei Lian Ma Huang Fang* | Eupatorii Herba (Pei lan), Coptidis Rhizoma (Huang lian), Ephedrae Herba (Ma huang) |
| Ping Gan Yi Shen Tiao Tan Yin | Gastrodiae Rhizoma (Tian ma), Uncariae Ramulus cum Uncis (Gou teng), Alismatis Rhizoma (Ze xie), Achyranthis Bidentatae Radix (Niu xi (chuan)), Atractylodis Macrocephalae Rhizoma (Bai zhu), Sargassum (Hai zao), Cassiae Semen (Jue ming zi), Taxilli Herba (Sang ji sheng), Pheretima (Di long), Prunellae Spica (Xiao ku cao), Pinelliae Rhizoma (Ban xia (zhi)), Crataegi Fructus (Shan zha) |
| Qiang Gan Jiao Nang | Artemisiae Scopariae Herba (Yin chen), Isatidis Radix (Ban lan gen), Codonopsis Radix (Dang shen), Dioscoreae Rhizoma (Shan yao), Astragali Radix (Huang qi), Angelicae Sinensis Radix (Dang gui), Salviae Miltiorrhizae Radix et Rhizoma (Dan shen), Curcumae Radix (Yu jin), Polygonati Rhizoma (Huang jing), Gentianae Macrophyllae Radix (Qin jiao), Alismatis Rhizoma (Ze xie), Glycyrrhizae Radix (Gan cao) |
| Qing Xue Dan | Scutellariae Radix (Huang qin), Coptidis Rhizoma (Huang lian), Phellodendri Chinensis Cortex (Huang bai), Gardeniae Fructus (Zhi zi), Rhei Rhizoma (Da huang) |
| Qu Tan Qing Wei Fang | Sophorae Flavescentis Radix (Ku shen), Phellodendri Chinensis Cortex (Huang bai), Chrysanthemi Indici Flos (Ye ju hua), Aurantii Fructus (Zhi shi (chao)), Gynostemmae Pentaphylli herba (Jiao gu lan), Nelumbinis Folium (He ye), Cassiae Semen (Jue ming zi (chao)), Gardeniae Fructus (Zhi zi (sheng)), Taraxaci Herba (Pu gong ying), Massa Medica Fermentata (Liu shen qu), Crataegi Fructus (Shan zha), Galli Gigerii Endothelium Corneum (Ji nei jin (chao)) |
| Qu Tan Tiao Zhi Tang | Salviae Miltiorrhizae Radix et Rhizoma (Dan shen), Notoginseng Radix et Rhizoma (Tian qi), Crataegi Fructus (Shan zha), Pinelliae Rhizoma (Ban xia (fa)), Citri Reticulatae Pericarpium (Chen pi), Poria cocos (Fu ling), Eucommiae Cortex (Duzhong (chao)), Astragali Complanati Semen (Sha yuan zi) |
| RCM-104 | Camellia Sinensis (Lü cha), Cassiae Semen (Jue ming zi), Sophorae Flos (Huai hua) |
| Shan Zha Xiao Zhi Jiao Nang* | Crataegi Fructus (Shan zha), Rhei Rhizoma (Da huang), Glycyrrhizae Radix (Gan cao) |
| Sheng Yang Li Shi Fang Ke Li Chong Ji | Ligustri Lucidi Fructus (Nü zhen zi), Coptidis Rhizoma (Huang lian), Alismatis Rhizoma (Ze xie), Nelumbinis Folium (He ye), Anemarrhenae Rhizoma (Zhi mu) |
| Target Herbal Ingredient | Scutellariae Radix (Huang qin), Platycodonis Radix (Jie geng) |
| Tian Mai Xiao Ke Pian | Schisandrae Chinensis Fructus (Wu wei zi), Ophiopogonis Radix (Mai dong), Trichosanthis Radix Tian hua fen) |
| TJ001 | Coicis Semen (Yi yi ren), Castaneae Semen (Gan li), Raphani Semen (Lai fu zi), Schisandrae Fructus (Wu wei zi), Liriopis tuber (Mai men dong), Ephedrae Herba (Ma huang), Platycodonis Radix (Jie geng), Acori Tatarinowii Rhizoma (Shi chang pu) |
| Wen Shen Jian Pi Hua Tan Fang | Poria cocos (Fu ling), Epimedii Folium (Yin yang huo), Citri Reticulatae Pericarpium (Chen pi), Atractylodis Macrocephalae Rhizoma (Bai zhu), Crataegi Fructus (Shan zha), Nelumbinis Folium (He ye) |
| Wu Ling San Jia Wei | Poria cocos (Fu ling), Atractylodis Macrocephalae Rhizoma (Bai zhu), Dioscoreae Rhizoma (Shan yao), Coicis Semen (Yi yi ren), Trichosanthis Radix (Tian hua fen), Alismatis Rhizoma (Ze xie), Polyporus (Zhu ling), Anemarrhenae Rhizoma (Zhi mu), Crataegi Fructus (Shan zha), Cinnamomi Ramulus (Gui zhi), Nelumbinis Folium (He ye), Glycyrrhizae Radix (Gan cao (sheng)) |
| Xie Re Hua Zhuo Fang | Coptidis Rhizoma (Huang lian), Scutellariae Radix (Huang qin), Polygoni Cuspidati Rhizoma et Radix (Hu zhang), Arnebiae Radix (Zi cao), Lonicerae Japonicae Flos (Yin hua), Atractylodis Rhizoma (Cang zhu), Nelumbinis Folium (He ye) |
| Hong He Qing Jiang Jiao Nang | Nelumbinis Folium (He ye), Atractylodis Rhizoma (Cang zhu), Crataegi Fructus (Shan zha), Pinelliae Rhizoma (Ban xia), Scutellariae Radix (Huang qin), Coptidis Rhizoma (Huang lian), Codonopsis Radix (Dang shen), Zingiberis Rhizoma (Gan jiang) |
| YY-312 | Imperatae Rhizoma (Bai mao gen), Citri Reticulatae Pericarpium (Chen pi), Euodiae Fructus (Wu zhu yu) |

Note: *This formula has been used in two different studies.

**
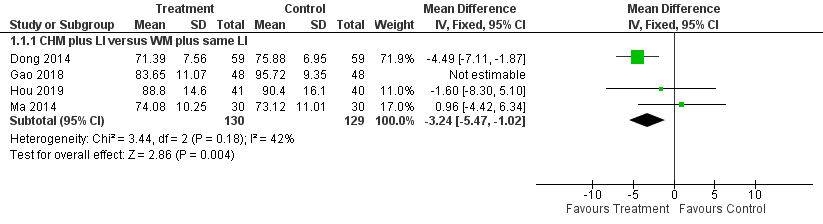
**

**Figure S1: Forest plot demonstrating the effects of CHM plus LI versus WM plus same LI on body weight after excluding outlying study.**

Abbreviations: CHM, Chinese herbal medicine; LI, lifestyle intervention; WM, Western medication

**
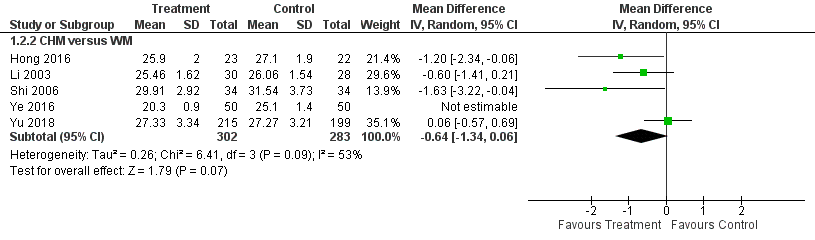
**

**Figure S2: Forest plot demonstrating the effects of CHM versus WM on body mass index after excluding outlying study.**

Abbreviations: CHM, Chinese herbal medicine; LI, lifestyle intervention; WM, Western medication
